# Chlorin e6-modified iron oxide nanoparticles for photothermal-photodynamic ablation of glioblastoma cells

**DOI:** 10.3389/fbioe.2023.1248283

**Published:** 2023-07-19

**Authors:** Hongqing Yao, Jian-Ying Zhou

**Affiliations:** Department of Nursing Care, Shanghai Songjiang District Central Hospital, Shanghai, China

**Keywords:** glioblastoma, iron oxide nanoparticles, photothermal therapy, photodynamic therapy, cancer therapy

## Abstract

**Introduction:** The effective treatment of glioblastoma still remains a great challenge. We herein report the development of chlorin e6 (Ce6)-conjugated iron oxide (Fe_3_O_4_-Ce6) nanoparticles for ablation of glioblastoma cells via combining photothermal therapy (PTT) with photodynamic therapy (PDT).

**Methods:** Ce6 was conjugated to the synthesized Fe_3_O_4_ nanoparticles to form Fe_3_O_4_-Ce6 nanoparticles displaying the optical property of Ce6.

**Results and discussion:** Under 808 nm laser irradiation, Fe_3_O_4_-Ce6 nanoparticles generated heat and the temperature increase did not have obvious changes after five cycles of laser irradiation, suggesting their good photothermal effect and photothermal stability. In addition, 660 nm laser irradiation of Fe_3_O_4_-Ce6 nanoparticles produced singlet oxygen (^1^O_2_) to mediate PDT. The Fe_3_O_4_-Ce6 nanoparticles without laser irradiation showed a low cytotoxicity, but they would obviously kill C6 cancer cells after laser irradiation via the combinational effect of PTT and PDT. Fe_3_O_4_-Ce6 nanoparticles thus could be used as a nanotherapeutic agent for combinational ablation of glioblastoma cells.

## 1 Introduction

Glioblastoma is the most common primary malignant tumor of the nervous system, accounting for about 40%–50% of all primary intracranial tumors (van Landeghem et al., 2009; Xin et al., 2012; Fang et al., 2015; Pinel et al., 2019). Because of the high degree of malignancy and short overall survival of glioblastoma patients, it is still a great challenge for the treatment of glioblastoma (Kuang et al., 2018; Choi et al., 2020; Gregory et al., 2020; Zhang et al., 2021; Wang et al., 2023). At present, the treatment of glioma is mainly based on surgery, which can be used to resect early small tumors in appropriate locations (Lara-Velazquez et al., 2017; Zhang et al., 2019a; De Witt Hamer et al., 2019). As the tumor grows due to its unclear boundaries, it is difficult to completely remove tumor cells (Wang et al., 2022; Dhar et al., 2022; Sandbhor et al., 2022; Zhang et al., 2023). Therefore, chemotherapy, radiotherapy, and immunotherapy have been used to combine surgery to further delay the progression of the disease and improve survival time (Zhang et al., 2019b; Ruan et al., 2019; Han et al., 2020; Lu et al., 2020; Alghamri et al., 2022; Wang et al., 2022; Li et al., 2022; Sun et al., 2022; Zhang et al., 2022; Zhang et al., 2023). However, due to short-term recurrence and drug resistance, the treatment effects of glioblastoma is not satisfactory.

Phototherapy is a type treatment strategy that relies on the light irradiation of tumors (Xie et al., 2020; Li et al., 2021; Zheng et al., 2021; Lee et al., 2022; Roy et al., 2023a). Compared to traditional chemotherapy, phototherapy shows the advantages of high selectivity, low side effects and negligible drug resistance (Cao et al., 2021; Huang et al., 2021; Pivetta et al., 2021; Feng et al., 2022). Photothermal therapy (PTT) utilizes the generated heat after laser irradiation of photothermal agents to ablate tumor cells (Fernandes et al., 2020; Gao et al., 2021; Lv et al., 2021; Huang et al., 2022). Photodynamic therapy (PDT) produces reactive oxygen species (ROS) to kill cancer cells via activating photosensitizers by light (Chen et al., 2020; Pham et al., 2021; Wan et al., 2021; Roy et al., 2023b). Currently, both PTT and PDT have been widely explored for treatments of different tumors. In addition, the combinations of PTT and PDT can lead to better efficacy for suppressing tumors (Curcio et al., 2019; Zhang et al., 2020; Chen et al., 2021).

In this study, we reported the development of chlorin e6 (Ce6)-conjugated iron oxide (Fe_3_O_4_-Ce6) nanoparticles for ablation of glioblastoma cells by PTT-combined PDT. Fe_3_O_4_ nanoparticles were first synthesized and their surface modification of Ce6 led to the formation of Fe_3_O_4_-Ce6 nanoparticles, in which, Fe_3_O_4_ nanoparticles and Ce6 were used as photothermal agents and photosensitizers, respectively. The morphology, hydrodynamic size, zeta potential, absorbance and fluorescence properties of Fe_3_O_4_-Ce6 nanoparticles were studied. Under 808 and 660 nm laser irradiation, Fe_3_O_4_-Ce6 nanoparticles could mediate PTT and PDT by generating heat and ROS. In addition, they were found to have a good photothermal stability after five cycles of laser irradiation. Via combining PTT and PDT, Fe_3_O_4_-Ce6 nanoparticles effectively killed C6 cells under 808 and 660 nm laser irradiation. Thus, Fe_3_O_4_-Ce6 nanoparticles could be used for ablation of glioblastoma cells via combinational therapy.

## 2 Materials and methods

### 2.1 Materials

Ce6 was purchased from America J&K Scientific Ltd. (United States). Bovine serum albumin (BSA), N-(3-dimethylaminopropyl)-N-ethyl-carbodiimide hydrochloride crystalline (EDC), N-hydroxysuccin-imide (NHS), singlet oxygen sensor green (SOSG), 2′,7′-dichlorodihydrofluorescein diacetate (H_2_DCFDA) and calcein-AM/propidium iodide (PI) double staining kit were purchased from Sigma Aldrich (United States). FeCl_3_.6H_2_O and FeCl_2_.4H_2_O were obtained from Aladdin Biochemical Technology Co., Ltd. (Shanghai, China). CCK-8 was purchased from Dojindo Laboratories (Japan). All the other chemicals were purchased from National Pharmaceutical Corporation (Shanghai, China).

### 2.2 Characterization techniques

Transmission electron microscopy (TEM) images were obtained using Tecnai G2 20 TWIN TEM (FEI, United States). Hydrodynamic sizes and zeta potential values were measured using a Zetasizer (Nano S90, UK). UV-vis absorptions were measured using persee UV-vis spectrophotometer (TU-1810, China). Fluorescence spectra were recorded using fluorescence spectrophotometer (Shimadzu RF-6000, Japan).

### 2.3 Synthesis of Fe_3_O_4_ nanoparticles

FeCl_2_.4H_2_O (89.0 mg) and FeCl_3_.6H_2_O (157.0 mg) were dissolved in 8.0 mL water, and then 5 mL aqueous solution containing NaOH (1.0 g) and BSA (20.0 mg) was dropped into above solution. The resulted solution was stirred at 80°C for 30 min and black products were formed. Then the solution was cooled to room temperature and the formed products were precipitated by using magnetic beads. After purification through water washing, BSA-coated Fe_3_O_4_ nanoparticles were obtained.

### 2.4 Synthesis of Fe_3_O_4_-Ce6 nanoparticles

Ce6 (12.0 mg), EDC (24.6 mg) and NHS (23.0 mg) were dissolved in 5 mL dimethyl sulfoxide and the solution were stirred at room temperature for 3 h. Then above solution was dropped into 5 mL solution of BSA-coated Fe_3_O_4_ nanoparticles, and the reaction was contained at room temperature for 24 h. The products were collected using magnetic beads and then further washed with water. After that, Fe_3_O_4_-Ce6 nanoparticles were obtained.

### 2.5 Evaluation of photodynamic efficacy

The solution of Fe_3_O_4_-Ce6 nanoparticles were mixed with SOSG, and the resulted solutions were irradiated by 660 nm laser (0.3 W/cm^2^) for different times. The fluorescence spectra of solutions without or with laser irradiation were recorded. The fluorescence intensities of solutions at 525 nm were used to evaluate the ^1^O_2_ generation by calculating the fluorescence enhancement (F/F_0_).

### 2.6 Evaluation of photothermal efficacy

The solutions of Fe_3_O_4_-Ce6 nanoparticles at different concentrations were irradiated by 808 nm laser (1.0 W/cm^2^), and the temperatures of solutions under laser irradiation were measured. To evaluate the photothermal stability, the nanoparticle solutions were irradiated by 808 nm laser (1.0 W/cm^2^) for five times and the temperatures of solutions were measured.

### 2.7 Evaluation of cell viability

The cell lines (brain endothelial bEnd.3 cells and rat C6 glioma cells) presents in this study were obtained from American Type Culture Collection (ATCC, United States). The bEnd.3 and C6 cancer cells were incubated with Fe_3_O_4_-Ce6 nanoparticles at different concentrations for 24 h, and then the cells were washed with PBS. The cells were then incubated in cell culture medium containing CCK-8 agent for 2 h. The supernatant of treated cells was collected to measure the absorbance at 450 nm using a Thermo Scientific Multiskan MK3 ELISA reader (Thermo scientific, United States), and then the cell viabilities were calculated.

### 2.8 Evaluation of therapeutic efficacy

C6 cancer cells were incubated with Fe_3_O_4_-Ce6 nanoparticles for 24 h and then the cells were irradiated by 808 nm laser (1.0 W/cm^2^) for 5 min and 660 nm laser (0.3 W/cm^2^) for 5 min. The cells were further incubated for 6 h and then the cell viabilities of cells were measured using CCK-8 analysis.

### 2.9 Calcein-AM/PI double staining

C6 cancer cells were incubated with Fe_3_O_4_-Ce6 nanoparticles for 24 h and then the cells were cultured in cell culture medium containing calcein-AM/PI double staining agent. The 808 nm laser (1.0 W/cm^2^, 5 min) and 660 nm laser (0.3 W/cm^2^, 5 min) was used to treat the cells. The fluorescence images of cells in various treatment groups were captured using a fluorescence microscope.

### 2.10 Intracellular ROS generation evaluation

C6 cancer cells were incubated with Fe_3_O_4_-Ce6 nanoparticles for 24 h and then the cells were further cultured in cell culture medium containing H_2_DCFDA for 30 min. The cells were then irradiated by 660 nm laser (1.0 W/cm^2^) for 5 min. Fluorescence images of cells in various treatment groups were captured using a fluorescence microscope.

### 2.11 Cellular uptake evaluation

C6 cancer cells were incubated with Fe_3_O_4_-Ce6 nanoparticles at different concentration for 12 h, and then the cells were washed with PBS to remove free nanoparticles. The contents of nanoparticles inside cells were evaluated by measuring intracellular Fe concentration using inductively coupled plasma optical emission spectroscopy (ICP-OES).

### 2.12 Statistical analysis

The data were provided as mean ± standard deviation (SD). Statistical analysis was carried out using one-way ANOVA statistical analysis. Statistical significance was indicated as (*) *p* < 0.05, (**) *p* < 0.01, and (***) *p* < 0.001.

## 3 Results and discussion

### 3.1 Characterization of Fe_3_O_4_-Ce6 nanoparticles

**FIGURE 1:**
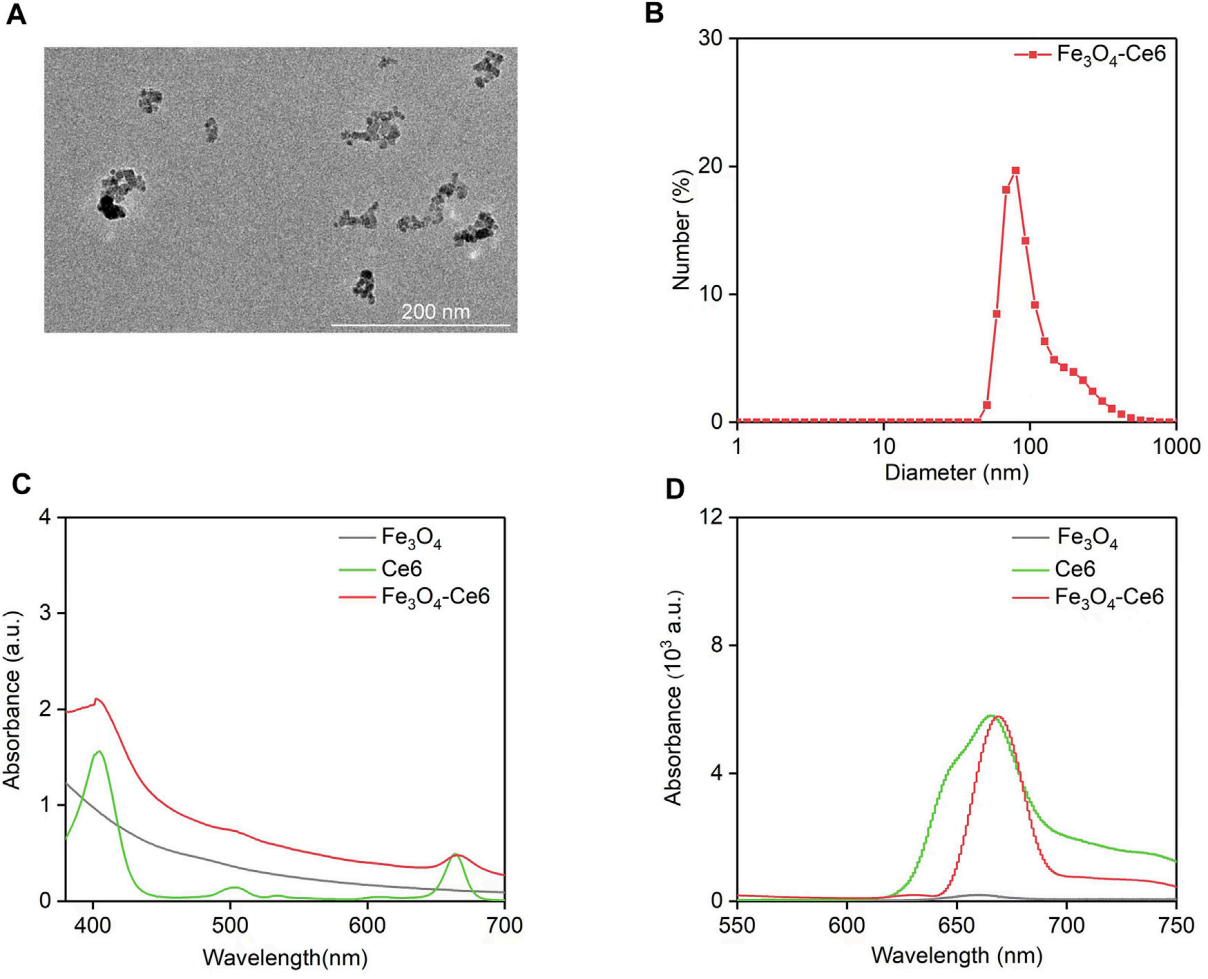
Characterization of Fe_3_O_4_-Ce6 nanoparticles. **(A)** TEM image of Fe_3_O_4_-Ce6 nanoparticles. **(B)** Hydrodynamic size of Fe_3_O_4_-Ce6 nanoparticles. **(C)** UV-Vis absorbance spectra of Fe_3_O_4_ nanoparticles, Ce6 and Fe_3_O_4_-Ce6 nanoparticles. **(D)** Fluorescence spectra of Fe_3_O_4_ nanoparticles, Ce6 and Fe_3_O_4_-Ce6 nanoparticles.

TEM image showed that the formed Fe_3_O_4_-Ce6 nanoparticles had a spherical morphology and their size distribution was homogeneous (Figure 1A). The hydrodynamic size and zeta potential of Fe_3_O_4_-Ce6 nanoparticles was measured to be 80.0 nm and −15.4 mV, respectively (Figure 1B). As shown in UV-vis spectra, the characteristic peaks of Ce6 at 400 nm and 641 nm could be detected in the absorbance spectrum of Fe_3_O_4_-Ce6 nanoparticles (Figure 1C), which however could not be detected in absorbance spectrum of Fe_3_O_4_ nanoparticles, confirming the conjugation of Ce6 to Fe_3_O_4_ nanoparticles. In addition, Fe_3_O_4_-Ce6 nanoparticles showed a fluorescence emission at around 670 nm (Figure 1D), and the fluorescence signal was also observed for Ce6. However, Fe_3_O_4_ nanoparticles did not have fluorescence property. These results suggested that Fe_3_O_4_-Ce6 nanoparticles showed the optical properties of Ce6.

### 3.2 Photothermal property of Fe_3_O_4_-Ce6 nanoparticles

**FIGURE 2:**
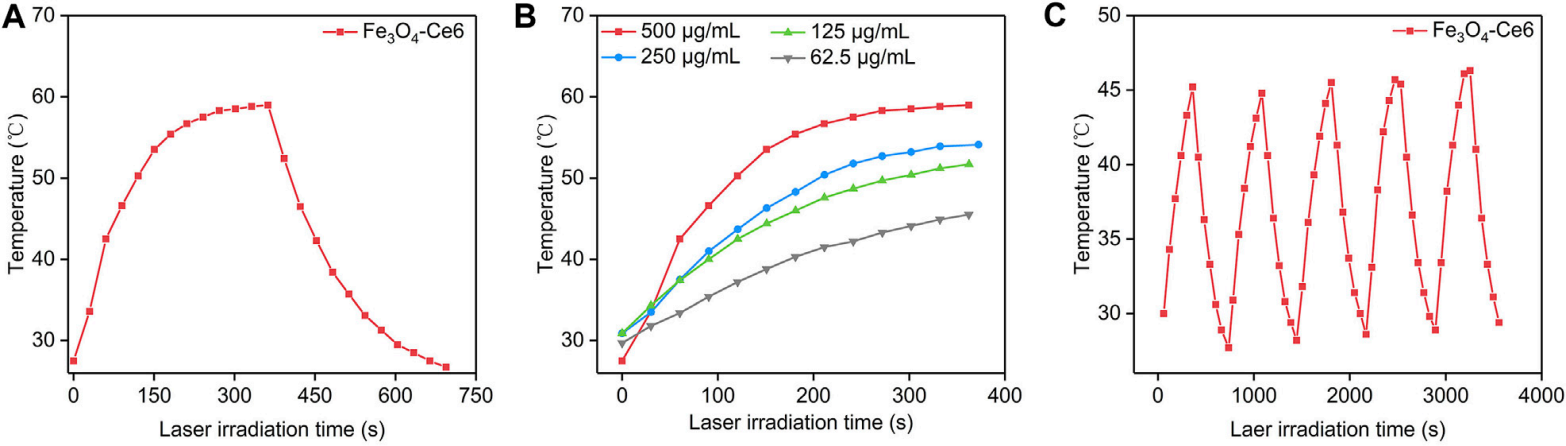
Photothermal property of Fe_3_O_4_-Ce6 nanoparticles. **(A)** Temperature changes of Fe_3_O_4_-Ce6 nanoparticles under 808 nm laser irradiation for laser on (6 min) and laser off (6 min). **(B)** Temperature increase for Fe_3_O_4_-Ce6 nanoparticles at different concentrations under 808 nm laser irradiation. **(C)** Temperature changes of Fe_3_O_4_-Ce6 nanoparticles after five cycles of laser on and laser off.

The photothermal property of Fe_3_O_4_-Ce6 nanoparticles under 808 nm laser irradiation was evaluated. Under laser irradiation, the temperature of solutions containing Fe_3_O_4_-Ce6 nanoparticles gradually increased, which reached around 58.8°C after 6 min of laser irradiation (Figure 2A). This result verified the good photothermal property of Fe_3_O_4_-Ce6 nanoparticles. The temperature increase for Fe_3_O_4_-Ce6 nanoparticles was found to be concentration-dependent, as higher concentration led to a higher temperature (Figure 2B). At the concentration of 500 μg/mL, the temperature increased to 58.8°C after 6 min of laser irradiation. In addition, the temperature increase did not have obvious changes after 5 cycles of laser on and laser off (Figure 2C). These results verified the good photothermal stability of Fe_3_O_4_-Ce6 nanoparticles. The good photothermal effect and good photothermal stability were similarly observed for Fe_3_O_4_ nanoparticles as reported in a previous study (Chen et al., 2023).

### 3.3 Photodynamic property of Fe_3_O_4_-Ce6 nanoparticles

**FIGURE 3:**
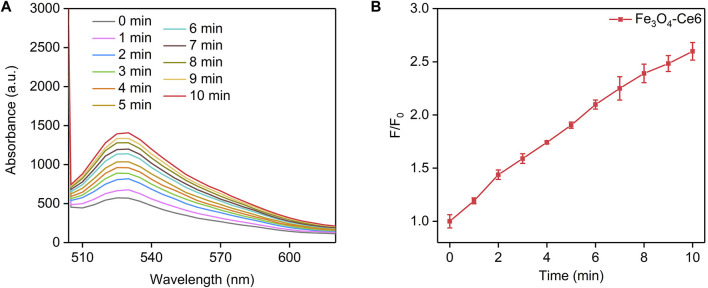
Photodynamic property of Fe_3_O_4_-Ce6 nanoparticles. **(A)** Fluorescence spectra of SOSG in solutions containing Fe_3_O_4_-Ce6 nanoparticles under 660 nm laser irradiation for different time. **(B)** Fluorescence changes of SOSG in solutions containing Fe_3_O_4_-Ce6 nanoparticles under 660 nm laser irradiation for different time.

The photodynamic property of Fe_3_O_4_-Ce6 nanoparticles was evaluated by measuring the generation of ^1^O_2_ under 660 nm laser irradiation using SOSG as the ^1^O_2_ probe. The fluorescence intensity of SOSG for solutions containing Fe_3_O_4_-Ce6 nanoparticles gradually increased under 660 nm laser irradiation (Figure 3A). This should be because the generated ^1^O_2_ turned on the fluorescence signals of SOSG. The fluorescence intensity of SOSG for solutions containing Fe_3_O_4_-Ce6 nanoparticles increased by 1.2-, 1.4-, 1.6-, 1.7-, 1.9-, 2.1-, 2.2-, 2.3-, 2.5-, and 2.6-fold after 1, 2, 3, 4, 5, 6, 7, 8, 9, and 10 min of laser irradiation (Figure 3B). These results confirmed the generation of ^1^O_2_ via photodynamic effect for Fe_3_O_4_-Ce6 nanoparticles under 660 nm laser irradiation. The ^1^O_2_ generating efficacy of Fe_3_O_4_-Ce6 nanoparticles was higher than that of protoporphyrin IX (PpIX)-modified Fe_3_O_4_ nanoparticles (Ding et al., 2022).

### 3.4 Cell viability and therapeutic efficacy evaluation

**FIGURE 4:**
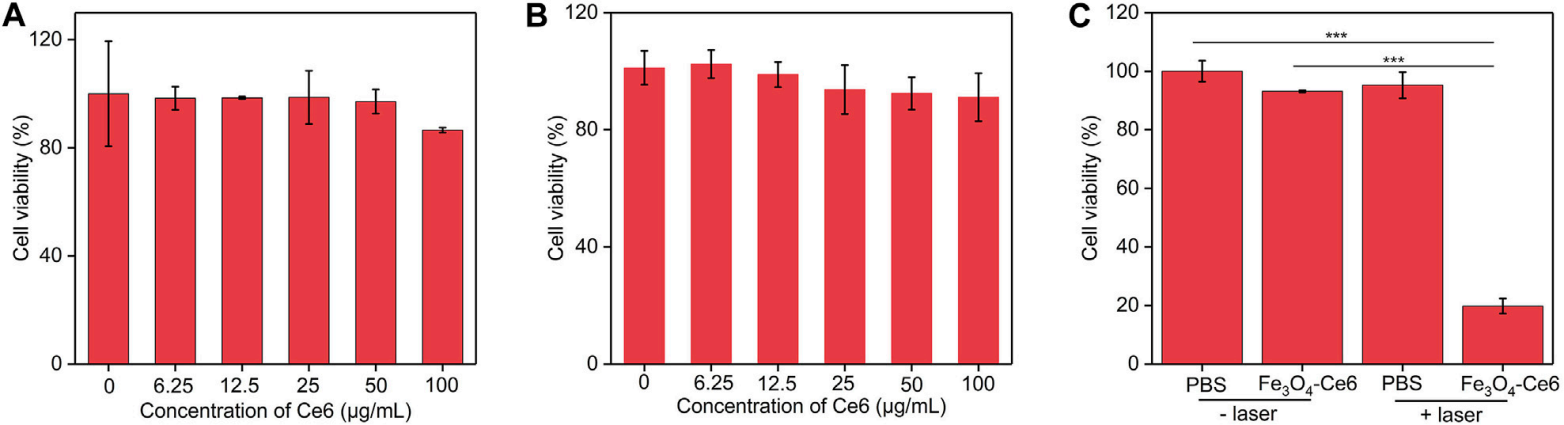
Cell viability and therapeutic efficacy evaluation. **(A)** Cell viability of bEnd.3 cells after incubation with Fe_3_O_4_-Ce6 nanoparticles at different concentrations for 24 h **(B)** Cell viability of C6 cells after incubation with Fe_3_O_4_-Ce6 nanoparticles at different concentrations for 24 h **(C)** Cell viability of C6 cells in PBS, PBS plus laser irradiation, Fe_3_O_4_-Ce6 nanoparticle treatment, and Fe_3_O_4_-Ce6 nanoparticle treatment plus laser irradiation groups.

To evaluate the cytotoxicity of nanoparticles to normal cells, bEnd.3 cells were incubated with these nanoparticles. After 24 h of incubation, the cell viability did not have obvious decline (Figure 4A). C6 cancer cells were incubated with Fe_3_O_4_-Ce6 nanoparticles at different concentrations for 24 h, and the CCK-8 analysis showed that the cell viability of these treated cells was still higher than 85.0% (Figure 4B), which suggested the low cytotoxicity for Fe_3_O_4_-Ce6 nanoparticles. To evaluate the *in vitro* therapeutic efficacy, C6 cells were incubated with Fe_3_O_4_-Ce6 nanoparticles and then treated by 808 and 660 nm laser. The cell viability for PBS + laser and Fe_3_O_4_-Ce6 nanoparticle-treated groups was similar to that in PBS control group (Figure 4C). These results suggested that laser irradiation and sole Fe_3_O_4_-Ce6 nanoparticle treatment did not have obvious therapeutic effect. In contrast, the cell viability of C6 cells in Fe_3_O_4_-Ce6 + laser group was only 19.6%, which suggested the good cell killing effect for Fe_3_O_4_-Ce6 nanoparticles plus laser irradiation via the combinational effect of PTT and PDT. The therapeutic efficacy of Fe_3_O_4_-Ce6 nanoparticles via PTT-combined PDT was higher than that of Fe_3_O_4_ nanoparticles via a sole PTT effect (Chen et al., 2023).

### 3.5 Dead/living staining analysis

**FIGURE 5:**
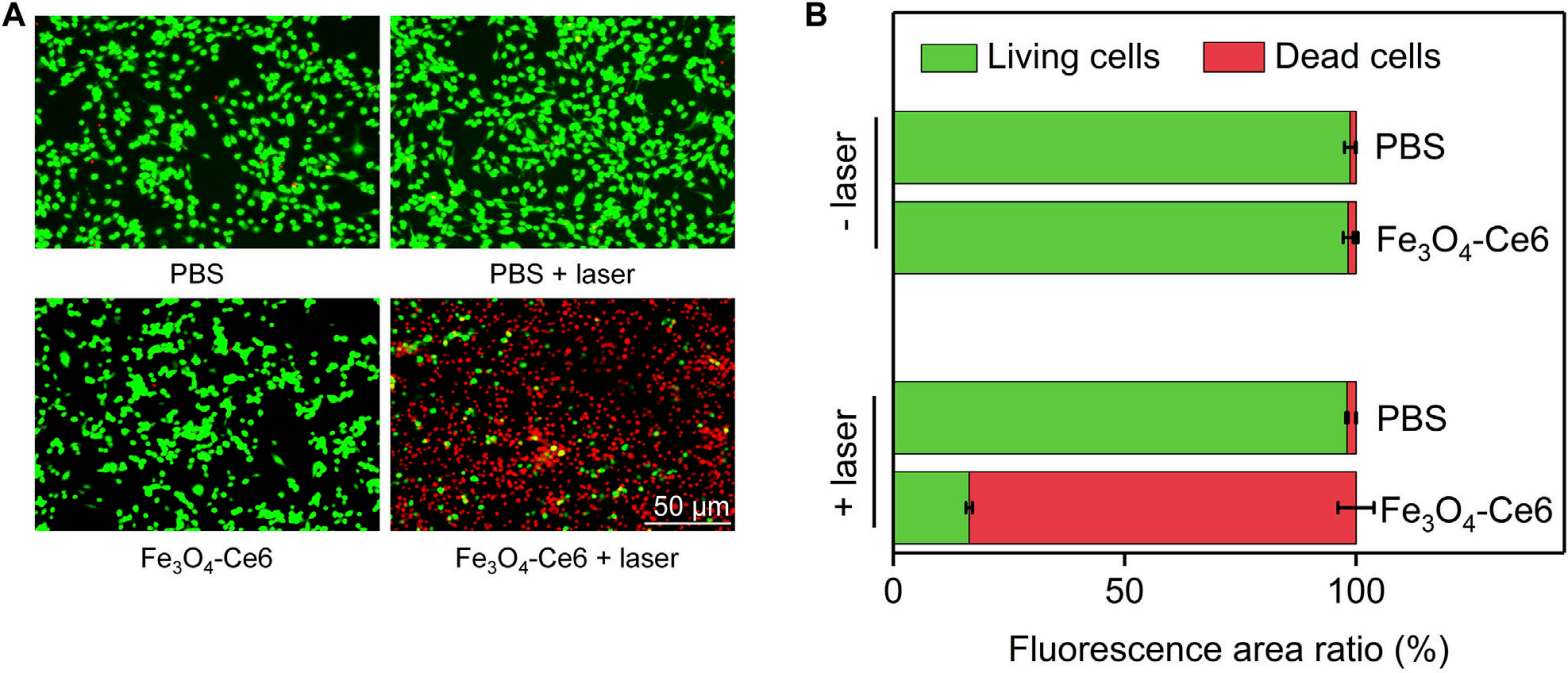
Dead/living staining analysis. **(A)** Dead and living fluorescence staining images of C6 cells after different treatments. **(B)** Quantitative analysis of the percentages of dead and living cells in different groups.

Dead/living staining was then used to evaluate the therapeutic efficacy. As shown in the fluorescence images, only green fluorescence signals (living cells) were observed for cells in PBS + laser and Fe_3_O_4_-Ce6 nanoparticle-treated groups, which was similar to those in PBS control group (Figure 5A). In contrast, both green and red fluorescence signals could be detected for cells in Fe_3_O_4_-Ce6 + laser group. The red fluorescence signals in this group indicated the death of cancer cells after treatment. Quantitative analysis showed that the percentage of dead cells and living cells in Fe_3_O_4_-Ce6 + laser group was 83.6% and 16.4%, respectively (Figure 5B). The percentages of living cells in PBS, PBS + laser and Fe_3_O_4_-Ce6 nanoparticle-treated groups were around 99.0%. These results further confirmed the good therapeutic efficacy for Fe_3_O_4_-Ce6 nanoparticles plus laser irradiation.

### 3.6 Intracellular ROS generation evaluation

**FIGURE 6:**
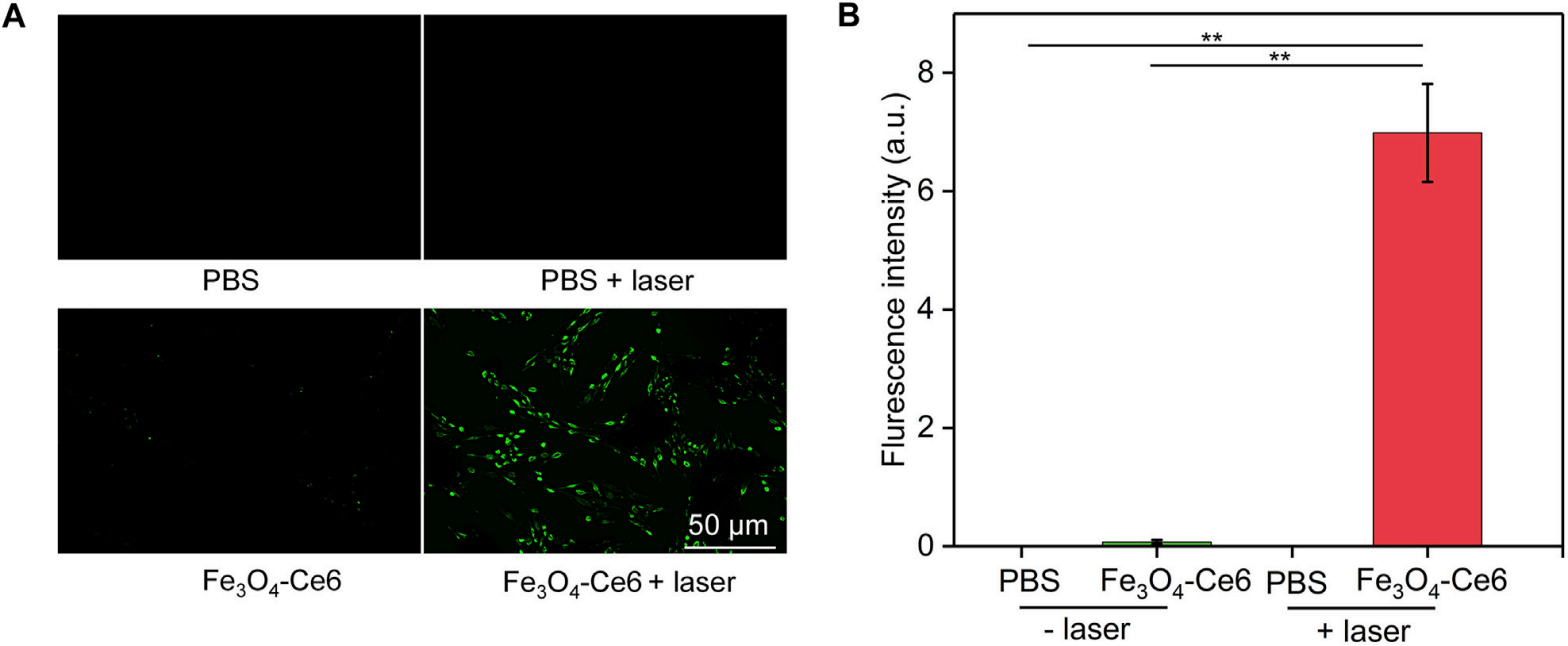
Intracellular ROS generation evaluation. **(A)** Fluorescence images of Ce6 cells in different groups to show the generation of ROS inside cells. **(B)** Quantitative fluorescence intensity of ROS in different groups.

To confirm the photodynamic effect, the generation of ROS inside cells after treatments was evaluated using H_2_DCFHDA as the ROS probe. Obvious green fluorescence signals could be detected in Fe_3_O_4_-Ce6 + laser group (Figure 6A), which verified the generation of ROS in this group. However, nearly no green fluorescence signals were observed in PBS and PBS + laser group. The very weak green fluorescence signal in Fe_3_O_4_-Ce6 nanoparticle-treated group may be due to the generation of a little ROS via Fenton reaction. The fluorescence intensity of green signals in Fe_3_O_4_-Ce6 + laser group was at least 82.0-fold higher than that in the other groups (Figure 6B). These results confirmed the generation of ROS inside cancer cells via photodynamic effect after Fe_3_O_4_-Ce6 nanoparticle treatment plus 660 nm laser irradiation.

### 3.7 Cellular uptake evaluation

**FIGURE 7:**
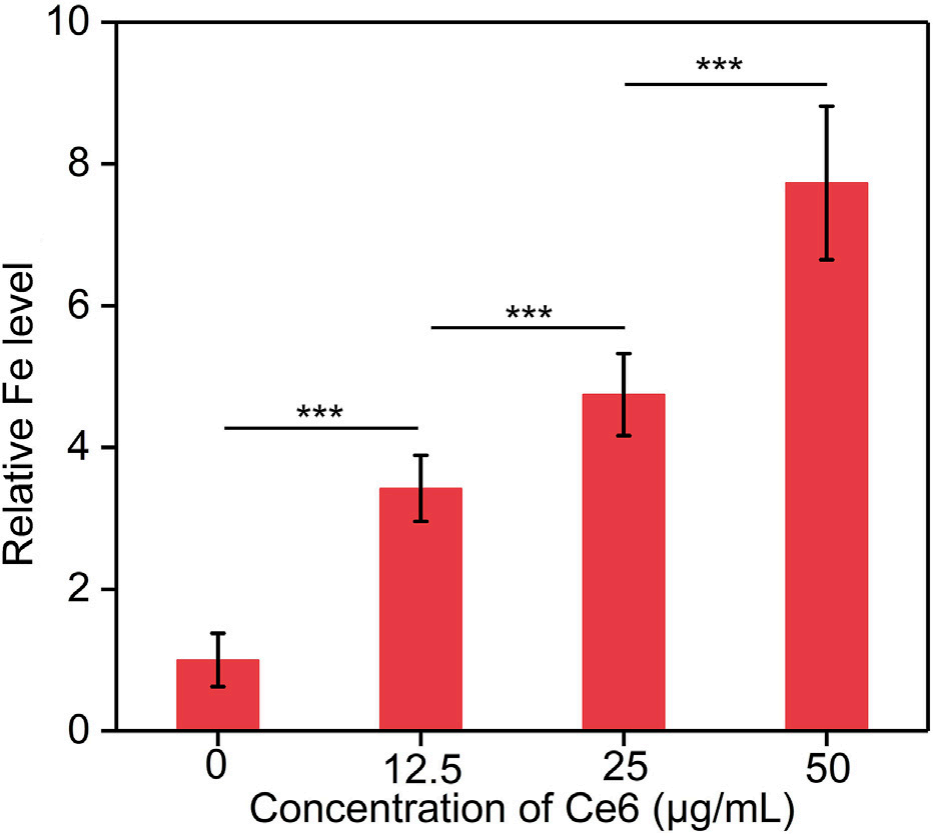
Cellular uptake analysis of Fe_3_O_4_-Ce6 nanoparticles by C6 cancer cells.

The cellular uptake of Fe_3_O_4_-Ce6 nanoparticles by C6 cancer cells were investigated. The results showed that the intracellular Fe levels in the treated cells gradually increased in a concentration depend manner (Figure 7). A higher concentration of nanoparticles led to a higher intracellular Fe level. These results confirmed the effective cellular uptake of Fe_3_O_4_-Ce6 nanoparticles by C6 cancer cells.

## 4 Conclusion

We have developed a nanoparticle system containing Ce6 and Fe_3_O_4_ nanoparticles for *in vitro* ablation of glioblastoma cells via combining PTT with PDT. Fe_3_O_4_-Ce6 nanoparticles were synthesized through conjugating Ce6 to Fe_3_O_4_ nanoparticles that showed negative surface potential and the optical property of Ce6. Fe_3_O_4_-Ce6 nanoparticles could mediate PTT and PDT via producing heat and ROS under 808 and 660 nm laser irradiation. The treatment of Fe_3_O_4_-Ce6 nanoparticles plus laser irradiation obviously killed cancer cells and reduced the cell viability, which were verified using CCK-8 analysis and living/dead staining. Fluorescence imaging confirmed the generation of ROS inside cancer cells for Fe_3_O_4_-Ce6 nanoparticle treatment plus laser irradiation. In view of the good fluorescence property of Fe_3_O_4_-Ce6 nanoparticles, they may be used for fluorescence imaging-guided combination therapy of cancer.

## Data Availability

The original contributions presented in the study are included in the article/supplementary material, further inquiries can be directed to the corresponding author.
